# Editorial: Invertebrate Neuroscience: Contributions From Model and Non-model Species

**DOI:** 10.3389/fnbeh.2021.726295

**Published:** 2021-07-14

**Authors:** Maria P. Fernandez, Clare C. Rittschof, Jimena A. Sierralta

**Affiliations:** ^1^Department of Neuroscience and Behavior, Barnard College of Columbia University, New York, NY, United States; ^2^Department of Entomology, College of Agriculture, Food, and the Environment, University of Kentucky, Lexington, KY, United States; ^3^Department of Neuroscience, Faculty of Medicine, Universidad de Chile, Santiago, Chile

**Keywords:** neurological diseases, aggression, addiction, social evolution, nutrition, *Drosophila*, bees

What is an animal model? Traditionally, models are used to investigate how humans develop, how our tissues and cells function, and how diseases take hold and progress. However, beyond these medical applications, many fields of biology, from evolution to neuroscience, also use model organisms to both identify foundational principles that unify species and to explore the extensive morphological and functional diversity among species. Although some vertebrates are powerful models due to their relevance to human physiology, they are of slow grow, expensive to breed and keep, and sometimes difficult to work with for *in vivo* experiments. On the other hand, invertebrates' forms, behaviors, sensory capabilities, and body organization are wonderfully diverse and unique, and yet these species also show surprising commonalities with other members of the animal tree of life. Neuroscientists are amazed that tiny brains can produce sophisticated behaviors with a limited number of neurons, that a butterfly can travel thousands of kilometers to rest for the winter, that even a tiny fly sleeps and wakes with a rhythm and that bees can communicate with a dance. This issue combines insights from traditional models such as *Drosophila melanogaster* and *Apis Mellifera* and organisms such as the crab *Neholice* and the cricket *Gryllus bimaculatus* to provide a glimpse into the power of invertebrate diversity to address fundamental and emerging questions in behavioral and comparative neuroscience.

The most widely used invertebrate model system in laboratory studies is *Drosophila melanogaster*, largely due to its unparalleled genetic tools that allow tissue or even cell specific manipulations of gene expression and neuronal activity, as well as a remarkably large collection of mutant and transgenic lines. *D. melanogaster* is used in a wide range of areas and applications in neuroscience, from molecular neuroscience to neurodevelopmental disorders and learning and memory studies. In this issue, several articles explore the utility of *D. melanogaster* in the context of human brain function and disease. McMullen et al. investigate the mechanism of sugar transport into the brain through the blood-brain barrier, a structure that protects the brain from harmful substances while allowing adequate nutrients to enter. The authors identify two previously unknown glucose transporters and perform functional analyses to demonstrate their critical role in survival. These findings are broadly relevant because of the extensive similarities between insect and mammalian brain nutrient transport. Carvajal-Oliveros and Campusano draw an even more direct link between *D. melanogaster* and human disease as they discuss the role of serotonin in neurodevelopmental disorders such as schizophrenia, autism, and attentional deficits; they describe the potential use of *D. melanogaster* to search for novel chemicals that could alleviate these diseases. A final area that highlights the links between *D. melanogaster* and human health is the neurobiology of addiction, reviewed in this issue by Chvilicek et al. The authors discuss recent progress in our understanding of the role of small-molecule neurotransmitters in alcohol response. They choose seven specific molecules that show functional conservation between mammals and other animal models, highlighting the use of *D. melanogaster* as a model for alcohol abuse.

Beyond human-oriented behavioral neuroscience, *D. melanogaster* research has significantly deepened our understanding of how genes and neuronal circuits control sexually dimorphic, innate behaviors such as courtship and aggression. Ryvkin et al. describe the transcriptome of neurons that express the fly homolog of the neuropeptide Y receptor (known as the neuropeptide F receptor), which plays a role in male courtship and social group interactions. The ultimate goal of this work is to understand how cell type specific gene expression shapes male behaviors. Sato and Yamamoto examine how changes in pheromone signaling and the sensory circuits underlying pheromone detection contribute to mate choice and reproductive isolation across *Drosophila* species. They focus on gustatory and non-volatile signals, as this type of chemosensory communication is key for species and sex recognition in *Drosophila*. Dietary restriction is a common environmental input that induces behavioral variation in animal species. Legros et al. show that *D. melanogaster* males raised on sugary diets attack rivals more frequently to establish dominance, employing fewer threat displays. *Drosophila*, and invertebrate species more generally, provide opportunities to examine both rapid and persistent environmental effects on behavior (Westwick and Rittschof).

Genetically tractable model systems such as *Drosophila* have proven extremely valuable for studying the underlying neuronal circuitry and the genetic architecture of complex behaviors under laboratory conditions. However, genetic models also have limitations, such as the lack of an ethological or ecological context. Pandolfi et al. compare genetic model systems and discuss their advantages and limitations for studying aggression. The authors discuss behavioral patterns and strategies observed in species such as *Homarus americanus* (lobsters) and *Gryllus bimaculatus* (crickets) for the study of aggressive behavior. The authors highlight a more general bias in the study of aggression, a focus on males, despite the importance of female aggressive behavior. Similarly, though *D. melanogaster* and *Apis mellifera* (honey bees) are very powerful models for the study of circadian rhythms, Beer and Helfrich-Förster argue that there are some aspects of chronobiology for which they are insufficient, such as investigating the role of the clock in photoperiodism and diapause. These authors advocate for the development of genetic tools in non-classical models, for example organism that exhibit a real photoperiodic diapause (such as the fruit fly *Drosophila triauraria* or the silkworm *Bombyx mori*), to enable studies of the diversity of biological clocks in insects, especially with respect to the timing of seasonal activity.

Several other contributors highlight interesting ways in which diverse invertebrate species, particularly insects, have the potential to contribute to outstanding questions in behavioral neuroscience. Though their biology can be strikingly different from vertebrates, contributors illustrate how these unique species can be leveraged to investigate questions that are broadly relevant. For example, Cámera et al. use the visually guided escape response in the crab *Neohelice granulata* to examine how distinct neurons act in concert to regulate a single behavior. They introduce a novel extracellular multi-electrode recording methodology that allows them to functionally distinguish previously described neurons, but also to identify novel neurons that do not fit in the known patterns, such as units sensitive to optic flow with directional preference. Muratore and Traniello explore the promise of using ants, specifically fungus-growing ants, to map the relationship between cognitive demand and brain structural evolution. Some species in this group contain several types of highly morphologically and behaviorally distinct sterile workers which show limited behavioral flexibility. Other related species exhibit less elaborate specialization with broad and flexible individual behavioral repertoires. Thus, it is possible through comparative study to examine the types of brain structural features that accompany behavioral specialization, and conversely, the features that are required to maintain cognitive flexibility. Westwick and Rittschof explore the potential for insects to contribute to our understanding of the link between early-life experience, neurobiology, and adult behavior. They highlight both the simple and complex environmental cues that give rise to adult behavioral variation in insects, and the diverse ways in which environmentally induced neurobiological changes are shared across insects and vertebrates. It could be debated to what extent unique insect systems are considered true “models;” however, it is clear that there is opportunity to use insects to study long-standing questions in behavioral neuroscience, some of which are less tractable in vertebrates.

In addition to their use as models and as the subjects of unique comparative studies, invertebrates present opportunities to tie behavioral neuroscience to broader scientific aims, for example conservation biology. Monarch butterfly seasonal migration presents a behavioral phenotype that is both unusual and generally relevant: monarchs are icons of pollinator conservation in the United States, and individuals are subject to unique challenges as they navigate over thousands of miles of mixed habitat. However, long-distance migration is a phenotype that is observed in diverse species including vertebrates. Guerra examines how monarchs integrate sensory cues from the environment to guide their long-distance navigation, a common challenge for migrating species, highlighting ways in which insights from the monarch may apply more broadly to animal migration and navigation. As with the monarch, the extent to which an invertebrate serves as a model for behavioral neuroscience is up to the willingness of the researcher to extend a comparative lens across a broad phylogenetic space.

The use of model and non-model invertebrates bring tremendous opportunity to explore behavioral neuroscience in a variety of laboratory and ecologically relevant contexts. The broad range of topics described in this issue, from the use of *Drosophila* for biomedical research to the fungus-growing ants to study changes in the brain associated to specific behaviors are examples of this.

## Author Contributions

All authors listed have made a substantial, direct and intellectual contribution to the work, and approved it for publication.

## Conflict of Interest

The authors declare that the research was conducted in the absence of any commercial or financial relationships that could be construed as a potential conflict of interest.

